# Author index

**DOI:** 10.1054/bjoc.2000.1351

**Published:** 2000-08-10


					Abel PD 59 (P124)
Aboagye E 30 (P6)
Abulafi AM 19 (4.7)
Achuthan R 82 (P216)
Adamson KL 18 (4.3)
Adeyemo D 58 (P117), 58
(P118)
Adlard JW 53 (P97), 65 (P148)
Affen J 41 (P52)
Agarwal R 28 (8.6), 80 (P208)
Ahern R 25 (7.5)
A'Hern R 24 (7.1), 41 (P50), 47
(P74), 50 (P88), 84 (P222)
A'Hern RP 2 (CT5)
Aherne GW 32 (P14)
Aherne W 26 (7.6)
Ahmed SI 78 (P198), 79 (P202)
Aitken M 54 (P102)
Alcock HE 82 (P214), 82
(P215)
Al-Husaini H 56 (P110)
Alison DL 79 (P204)
Allen D 49 (P83)
Allen JE 82 (P215)
Allen S 53 (P99)
Allen-Mersh TG 12 (1.6), 45
(P67)
Allison S 23 (6.2)
Allott CP 65 (P147)
Almond J 1 (CT1)
Al-Mufti RAM 51 (P90)
Al-Othman SF 56 (P110)
Al-Sakkaf KA 67 (P154)
Ameri K 11 (1.1), 11 (1.2)
Amess JAL 16 (3.4), 84 (P221)
Amirghofran Z 34 (P24)
Anderson A 37 (P35), 39 (P42)
Anderson D 71 (P169)
Anderson L 49 (P82)
Andreyev HJN 13 (2.1)
Anglian Breast Cancer Study
Group 36 (P31)
Anlezark GM 70 (P167)
Annink C 25 (7.3)
Ansell W 74 (P182)
Appanna TC 75 (P188)
Applebaum F 22 (5.7)
Arimidex First Line Study
Group 39 (P43)
Ash D 6 (S16)
Ashley S 25 (7.2), 43 (P60)
Ashworth A 25 (7.4)
Askaa J 37 (P33)
Assersohn L 25 (7.2), 43 (P57)
Athow ACA 41 (P51)
Atkin W 45 (P65)
Aukland A 2 (CT6)
Azim-Araghi A 65 (P145)
Babb P 17 (3.9)
Back JMV 25 (7.3)
Baddeley H 12 (1.7), 32 (P15)
Baddeley J 71 (P169)
Baguley BC 69 (P163)
Baird E 44 (P61)
Baker MB 69 (P164)
Ball HM-L 38 (P37)
Ball KL 15 (2.8)
Banerji U 26 (7.6)
Banker DE 22 (5.7)
Baokbah TA 23 (6.4)
Barclay AN 35 (P25)
Barlow R 16 (3.3)
Barnes D 15 (2.10)
Barnes DM 38 (P38)
Barnett G 73 (P180)
Barnwell J 1 (CT3)
Barrett-Lee PJ 49 (P83)
Bartlett J 37 (P35)
Bartlett JMS 38 (P39), 76
(P190)
Barton R 71 (P169)
Bass R 43 (P57)
Bassi C 1 (CT1)
Bate TE 56 (P109)
Bates N 78 (P200)
Batt LA 2 (CT8)
Bavetsias V 67 (P156)
Baxter J 44 (P62)
Beath S 72 (P175)
Bedford JL 74 (P183), 74
(P184)
Begent RHJ 11 (1.4), 13 (1.9),
18 (4.3), 29 (P4), 32 (P16), 71
(P170), 71 (P171), 71 (P172),
72 (P173)
Beger HG 1 (CT1)
Belelli D 57 (P115)
Bell SM 82 (P216)
Benghiat A 42 (P55)
Benson C 31 (P12)
Benson RJ 52 (P96), 75 (P185)
Bentzen SM 18 (4.1)
Berney DM 34 (P21), 76 (P189)
Berns A 5 (S11)
Bertwistle D 25 (7.4)
Betts CD 77 (P193)
Beynon T 50 (P85), 50 (P86)
Bhatia J 71 (P170), 71 (P172)
Bibby MC 23 (6.4), 63 (P137),
69 (P161), 69 (P162)
Bicknell R 30 (P6)
Bilger A 5 (S9)
Bing C 81 (P212)
Bingham S 45 (P65)
Bingham SA 81 (P209)
Bishop HM 2 (CT6)
Bissett D 51 (P92)
Blake PR 80 (P206)
Blakey D 31 (P9)
Blaydes JP 38 (P37)
Blesing C 18 (4.2)
Blunt RJ 2 (CT6)
Boddy AV 65 (P146), 79 (P203)
Boden R 13 (1.9), 32 (P16), 34
(P22)
Bond SJ 18 (4.1)
Bootle DE 69 (P164)
Bottomley S 82 (P215)
Boudioni M 53 (P100)
Bouer M 20 (4.10)
Boukamp P 9 (S25)
Boulos PB 46 (P72)
Boulton M 53 (P100)
Boxall F 65 (P147)
Boxer G 13 (1.9), 71 (P170)
Boxer GM 11 (1.4), 29 (P4)
Boyce LM 19 (4.6)
Boyd MT 60 (P125)
Bozcuk H 43 (P59)
Bradley C 63 (P137)
Bradshaw A 84 (P223)
Brady F 30 (P6)
Branston L 2 (CT8)
Briggs T 79 (P202)
Britten K 16 (3.3)
Bromelow KV 26 (7.8)
Brookes J 48 (P79)
Brooks R 72 (P174)
Brown BL 67 (P154)
Brown D 30 (P6)
Brown J 28 (8.7)
Brown MH 35 (P25)
Brown R 48 (P80)
Brown RSD 53 (P99), 54
(P102), 75 (P186)
Brown S 28 (8.7)
Brown T 23 (6.2), 44 (P62)
Brunt AM 51 (P91)
Brunton L 32 (P14)
Bryant P 39 (P41)
Bryce L 55 (P106)
Buchler MW 1 (CT1)
Bulmer J 34 (P22)
Bulusu VR 51 (P89), 66 (P149)
Bunce M 75 (P187)
Burch N 54 (P101)
Burchill SA 33 (P20), 58 (P119)
Burcombe RJ 37 (P36)
Burdett S 2 (CT7)
Burgess LA 80 (P207)
Burke B 11 (1.1), 11 (1.2)
Burke PJ 70 (P166)
Burnet NG 33 (P19), 52 (P96),
75 (P185)
Burrell AD 49 (P81)
Burton A 27 (8.3)
Burton H 52 (P93)
Burton KE 75 (P185)
Butler J 66 (P150), 66 (P152)
Cairns C 23 (6.2)
Caldas C 23 (6.3)
Calvert AH 79 (P203)
Cameron D 37 (P35)
Cameron DA 39 (P42), 61
(P129)
Campbell I 78 (P199)
Campbell J 72 (P176)
Campbell L 57 (P115)
Camplejohn R 15 (2.10)
Camplejohn RS 38 (P38), 61
(P130)
Cannell H 34 (P24)
Cao T 8 (S23)
Carder PJ 37 (P33)
Carey FA 44 (P64)
Carmichael J 11 (1.3), 21 (5.2),
55 (P105), 73 (P180)
Carnochan P 12 (1.6), 16 (3.2)
Carpenter E 16 (3.4)
Carpenter R 41 (P49), 41 (P51)
Carrington B 16 (3.3)
Carroll VA 30 (P6)
Carter M AZS 84 (P221)
Casanova MLL 8 (S24)
Case MC 64 (P143)
Cassidy J 19 (4.6), 35 (P28), 45
(P66), 68 (P157)
Catovsky D 16 (3.2)
Cavalli F 15 (3.1)
Chan CMW 62 (P135)
Chaplin DJ 31 (P10)
Charlton P 69 (P163)
Charlton PA 69 (P164)
Charlton RS 15 (2.10)
Chatterjee R 40 (P45)
Chaudhary KS 59 (P124)
Chester K 71 (P170)
Chester KA 11 (1.4), 71 (P171),
71 (P172)
Chinegwundoh F 74 (P182)
Choudry GA 63 (P137)
Chow S 20 (4.10)
Choy C 41 (P49), 41 (P51)
Chresta CM 56 (P109), 63 (P140)
Chu CE 15 (2.10)
Chumas P 15 (2.10)
Chung-Faye G 71 (P169)
Clark J 71 (P169)
Clark LF 38 (P39)
Clark PI 1 (CT2), 26 (7.7), 42
(P56)
Clarke AR 14 (2.7)
Clarke NW 77 (P193)
Clarke PA 13 (2.1)
Clayton K 55 (P105)
Cleator S 33 (P18)
Clelland C 73 (P179)
Clingen PH 63 (P138)
Clinton S 32 (P14)
Clive S 35 (P27)
Clutterbuck R 16 (3.2), 72
(P174)
Coe N 66 (P150)
Cohen BB 25 (7.3), 36 (P32)
Coleman JM 78 (P198)
Coleman R 28 (8.7), 67 (P155)
Coleman RE 78 (P198), 79
(P202)
Collette L 27 (8.5)
Collie D 52 (P95)
Author index
85
British Journal of Cancer (2000) 83(Suppl 1), 85-90
(c) 2000 Cancer Research Campaign
doi: 10.1054/ bjoc.2000.1351, available online at http://www.idealibrary.com on
Collie-Duguid ESR 19 (4.6)
Connor F 25 (7.4)
Conray S 42 (P55)
Conseiller E 24 (6.7)
Convery D 26 (8.1)
Cook G 72 (P176)
Cook PA 27 (8.5)
Cooke PW 27 (8.3)
Cooke SP 11 (1.4)
Cooke TG 38 (P39)
Cooley J 41 (P52)
Coombes RC 14 (2.5)
Cooper R 46 (P70)
Copeland L 57 (P115)
Copie-Bergmann C 15 (3.1)
Corbo M 22 (5.7)
Corfe B 20 (5.1)
Cormier R 5 (S9)
Cornbleet MC 61 (P129)
Corrie P 18 (4.2)
Cosgrove V 26 (8.1)
Costello E 59 (P123)
Cotter FE 22 (5.7), 22 (5.8), 64
(P144)
Cottrill C 33 (P18)
Coulson JM 42 (P53)
Coulthard S 64 (P142)
Coward D 77 (P195)
Cox J 49 (P82)
Craddock C 16 (3.5)
Cragg MS 58 (P120), 59
(P121)
Crawford DH 15 (2.9), 72
(P175)
Cree IA 80 (P205)
Cresswell J 73 (P178)
Crichton DN 38 (P40)
Croft J 44 (P62), 75 (P188)
Crook T 15 (2.9)
Cross AJ 81 (P209)
Crosse BA 54 (P103)
Croucher P 67 (P155)
Cummings J 35 (P27)
Cunningham D 1 (CT4), 13
(2.1), 19 (4.5)
Curling G 54 (P101)
Curran S 45 (P66)
Curry B 31 (P11)
Dabare AANPM 77 (P194)
Daft EL 13 (2.2)
Daley F 37 (P36)
Danenberg K 19 (4.5)
Danenberg P 19 (4.5)
Dangerfield W 69 (P163), 69
(P164)
Darby A 46 (P69)
Dassu D 28 (8.7)
D'Ath S 18 (4.2)
Davey J 54 (P101)
David G 29 (P1)
Davidson BR 22 (5.8), 64 (P144)
Davies BR 34 (P22), 34 (P23)
Davies EJ 29 (P1)
Davies MM 12 (1.6)
Davis PD 31 (P9), 31 (P10)
Day N 45 (P65)
de Mulder PHM 27 (8.5)
de Neve W 6 (S15)
de Prijck L 27 (8.5)
De Silva IU 63 (P138)
de Wit R 27 (8.5)
Deakin D 16 (3.3)
Dearling J 13 (1.9), 32 (P16)
Dearling JLJ 72 (P173)
Dearnaley D 26 (8.1)
Dearnaley DP 74 (P181), 74
(P183), 74 (P184), 77 (P195),
77 (P196), 78 (P197)
Debussche L 24 (6.7)
Decatris MP 42 (P55)
Delchier JC 15 (3.1)
Denholm E 72 (P173)
Denholm EE 63 (P138)
Denny WA 69 (P163), 69
(P164)
Deplanque G 78 (P200)
Dervernis C 1 (CT1)
Deshmane VH 55 (P107)
Dex E 62 (P134)
Di Francesco AM 66 (P152)
di Stefano F 13 (2.1)
Dias C 65 (P146)
Dickson J 47 (P76)
Dische S 48 (P77)
Dive C 20 (5.1)
Dobson PRM 67 (P154)
Dodson L 2 (CT6)
Dodwell DJ 39 (P44)
Donald S 81 (P211)
Dornan D 60 (P126)
Dorudi S 28 (8.8)
Double JA 69 (P161), 69
(P162)
Dougherty GJ 31 (P10)
Doughty JC 30 (P5), 40 (P47)
Douglas S 31 (P9)
Dove W 5 (S9)
Downey M 44 (P63)
Downing I 72 (P176)
Dowsett M 25 (7.2), 25 (7.5)
Droufakou S 55 (P107)
D'Souza D 46 (P72)
Duchesne GM 27 (8.2), 75
(P186)
Ducki S 67 (P153)
Duddy PM 38 (P38)
Dukes M 31 (P11)
Duncan P 76 (P190)
Dunlop DJ 42 (P54)
Dunn JA 1 (CT1), 2 (CT6)
Dyer MJS 16 (3.2), 72 (P174)
Dzik-Jurasz ASK 20 (4.8)
Easton DF 25 (7.2)
Ebbs S 2 (CT5)
Eccles D 78 (P199)
Eccles S 16 (3.2), 19 (4.7), 57
(P115)
Eccles SA 20 (4.8), 29 (P3)
Edmondson RJ 34 (P22), 34
(P23)
Edwards J 76 (P190)
Eeles R 24 (7.1), 47 (P74)
Eeles RA 25 (7.2)
Eisen T 31 (P12), 61 (P131)
El Baz M 59 (P124)
Elias D 47 (P73)
Ellis DJ 2 (CT6)
Englefield P 78 (P199)
Erenpreisa Je 58 (P120), 59
(P121)
European Osteosarcoma
Intergroup 18 (3.10)
Evans DG 41 (P52)
Eves P 62 (P133)
Farrugia D 20 (4.9), 47 (P73),
74 (P182)
Farrugia DC 19 (4.5)
Fashola-Stone E 70 (P167)
Felton-Edkins Z 23 (6.2)
Fennell DA 22 (5.7), 22 (5.8)
Fentiman IS 55 (P107)
Fernandez-Cruz L 1 (CT1)
Ferry D 71 (P169)
Fersht N 84 (P222)
Finan P 46 (P70)
Fischer P 68 (P158)
Fishwick K 79 (P203)
Flanagan E 78 (P200)
Flannigan GM 63 (P137)
Fletcher-Monaghan AJ 44 (P63)
Flynn AA 13 (1.9), 32 (P16), 72
(P173)
Foley R 16 (3.3)
Foot NJ 16 (3.4)
Ford HER 19 (4.5)
Foskett M 80 (P208)
Fossa SD 27 (8.5)
Foster RE 63 (P140)
Francis RJ 18 (4.3)
Frazer-Williams M-J 24 (7.1),
47 (P74)
Freiss H 1 (CT1)
Fresco L 42 (P55)
Fridman J 4 (S7)
Gabra H 24 (6.6)
Gabriel A 25 (7.4)
Gaffney CC 49 (P83)
Galanis A 6 (S13)
Galbraith SM 12 (1.7), 31 (P10)
Gale J 78 (P199)
Gallacher-Horley B 24 (6.8)
Gallagher C 20 (4.9)
Gallagher J 29 (P2)
Gallagher JT 29 (P1)
Gallagher WM 24 (6.7)
Ganesan R 27 (8.3)
Ganesan TS 78 (P200)
Ganta S 23 (6.4), 63 (P137)
Garner C 57 (P113), 57 (P114)
Gaukroger K 30 (P8)
Gayther SA 23 (6.3)
Geater AR 33 (P19)
Geh JI 18 (4.1), 53 (P98)
George J 78 (P198)
George M 13 (2.1), 19 (4.7)
George ML 20 (4.8)
George WD 30 (P5), 40 (P47)
Gerrard G 53 (P97)
Gerrard M 56 (P111)
Gescher A 20 (4.10), 24 (6.8),
81 (P211)
Ghaneh P 13 (2.3)
Gibson A 52 (P95)
Gilchrist R 61 (P130)
Gilhooley A 77 (P193)
Giridharan S 53 (P98)
Glaholm J 47 (P75)
Glaholm JG 76 (P192)
Glaser M 30 (P6)
Glennie MJ 17 (3.7)
Glioma Meta-analysis Trialists
Group 2 (CT7)
Glover C 45 (P67)
Glynne-Jones R 18 (4.1), 46
(P71)
Go C 8 (S23)
Goddard P 32 (P14)
Goddard PM 68 (P158)
Goepel JR 56 (P110), 82 (P214),
82 (P215)
Going JJ 44 (P63), 76 (P190)
Gomm JJ 14 (2.5)
Goodchild KA 12 (1.5)
Goodman A 47 (P75)
Gore M 61 (P129)
Gore ME 31 (P12)
Gould K 5 (S9)
Graham CF 35 (P25)
Graham J 52 (P95)
Graham JD 27 (8.2)
Grant R 52 (P95)
Gray R 1 (CT3)
Green AJ 18 (4.3)
Green J 14 (2.6)
Greenall A 6 (S13)
Greene LM 24 (6.7)
Greenhalf W 13 (2.3)
Greenhalgh R 41 (P52)
Gregory KR 26 (7.8)
Gregory RK 43 (P60)
Gregory W 1 (CT4)
Grieve RJ 2 (CT6)
Griffin MJ 79 (P203)
Griffiths G 20 (5.1)
Griffiths GO 27 (8.2)
Griffiths P 44 (P62)
Groeger G 35 (P25)
Guerrero Urbano T 76 (P191)
Gui G 41 (P50), 54 (P101)
Gui GPH 62 (P135)
Gul LA 67 (P153)
Gunter M 45 (P65)
Gurney E 28 (8.7)
Gusterson B 25 (7.2)
Gyselman V 28 (8.8)
Hadfield JA 30 (P7), 30 (P8)
Hadjiyiannakis P 16 (3.2), 72
(P174)
Haigis K 5 (S9)
Halberg R 5 (S9)
Haldar N 75 (P187)
Hale G 16 (3.2), 72 (P174)
Hale J 65 (P146)
Hall A 64 (P142), 72 (P174)
Hall AG 17 (3.6), 64 (P141), 64
(P143)
Hall CN 81 (P210)
Hall E 49 (P84)
Hall G 28 (8.7), 50 (P87)
Halstead F 68 (P159)
Hammond DW 82 (P214), 82
(P215)
Hampel C 23 (6.5)
Hanby AM 38 (P38), 55
(P107)
86 Author index
Hancock B 1 (CT4), 28 (8.7), 61
(P129)
Hancock BW 15 (3.1), 82
(P214), 82 (P215), 83 (P218),
83 (P219)
Hancock H 83 (P218), 83
(P219)
Hankin SL 56 (P111)
Hannah A 26 (7.6)
Hannigan K 26 (7.7)
Haque T 72 (P175)
Hardcastle A 26 (7.6), 32 (P14)
Harden G 72 (P174)
Hargreaves R 66 (P150)
Hargreaves RHJ 66 (P152)
Harland SJ 28 (8.6)
Harmer C 33 (P18), 50 (P88),
84 (P222)
Harper-Wynne C 25 (7.5)
Harrer E 50 (P87)
Harris A 11 (1.2), 75 (P187)
Harris AL 11 (1.1), 30 (P6), 37
(P34)
Harris M 16 (3.3)
Harris WH 55 (P107)
Harrison KL 81 (P210)
Hart I 36 (P30)
Hart IR 55 (P107), 61 (P130),
61 (P132), 62 (P134)
Hartley A 51 (P91), 53 (P98)
Hartley J 72 (P173)
Hartley JA 16 (3.5), 22 (5.6), 23
(6.5), 63 (P138), 63 (P139), 66
(P152), 70 (P165)
Harvey M 46 (P69)
Hassan AB 35 (P25), 35 (P26)
Hassanally D 51 (P90)
Hastings DL 30 (P7)
Hatton MQ 78 (P198)
Haward R 40 (P46)
Hayes L 41 (P52)
Hayne D 46 (P72)
Haynes R 28 (8.6)
Hazrati A 66 (P152)
He W 8 (S23)
Hejaz HAM 56 (P112)
Hellmann K 17 (3.8)
Henderson DC 45 (P67)
Hennequin LF 31 (P11)
Henry A 39 (P44)
Hewett PW 11 (1.3), 13 (2.2)
Hickish T 2 (CT5)
Hickman J 20 (5.1)
Hickman JA 56 (P109)
Higgins J 79 (P202)
Highley M 79 (P203)
Hill S 42 (P54)
Hill SA 12 (1.5), 13 (1.9), 31
(P10)
Hilson AJ 18 (4.3)
Hoare SF 55 (P106)
Hochhauser D 18 (4.3), 19 (4.4),
22 (5.6), 23 (6.5), 70 (P165)
Hockey A 63 (P138)
Hogarth L 64 (P142)
Hogarth LA 64 (P141)
Hogben K 54 (P101)
Holder AL 31 (P9)
Honeychurch J 17 (3.7)
Hope-Stone LD 18 (4.3)
Hopper C 48 (P79)
Horgan K 82 (P216)
Horsman J 83 (P218), 83 (P219)
Horsman JM 78 (P198)
Horwich A 16 (3.2), 72 (P174),
74 (P181), 77 (P195), 77
(P196), 78 (P197)
Hoskin P 1 (CT4), 32 (P15)
Hoskin PJ 12 (1.5), 27 (8.4), 43
(P58), 84 (P223)
Hough RE 82 (P214), 82 (P215)
Houlston R 24 (7.1), 47 (P74)
Hourihan RN 44 (P61)
Howell A 2 (CT5), 41 (P52)
Howell JA 35 (P26)
Howells L 20 (4.10)
Howie J 57 (P115)
Huang A 45 (P67)
Huddart R 74 (P181)
Huddart RA 77 (P195), 77
(P196), 78 (P197)
Hummerston S 55 (P105)
Humphreys M 13 (2.3)
Hunkler IH 25 (7.3)
Hunter JAA 61 (P129)
Hunter N 72 (P176)
Hupp TR 15 (2.8), 38 (P37), 60
(P126)
Hurman D 52 (P95)
Hussain NB 23 (6.4)
Hussain SA 76 (P192)
Hutton J 48 (P80)
Illidge TM 17 (3.7), 58 (P120),
59 (P121)
Ingham-Clark C 51 (P90)
Ingram A 15 (2.8)
Innes HE 42 (P56)
Innes J 72 (P176)
Ireson CR 81 (P211)
Islam-Ali B 82 (P213)
Ivanov A 58 (P120)
Ives F 66 (P149)
Iveson AM 46 (P69)
Iveson TJ 46 (P69)
Jackman AL 19 (4.5), 67 (P156)
Jacks T 5 (S10)
Jackson DL 49 (P81)
Jackson S 27 (8.4), 48 (P78)
Jacobs I 28 (8.8)
Jaeger R 48 (P79)
Jaffrey RG 35 (P28)
Jagdev S 67 (P155)
Jahan MA 52 (P93)
Jakobsen A-M 73 (P180)
James ND 27 (8.3), 76 (P192)
James R 19 (4.4)
James S 75 (P188)
Jan H 16 (3.3)
Jankowska PJ 17 (3.9)
Jarvis MC 23 (6.4)
Jay G 77 (P195)
Jayson G 29 (P1)
Jefferies S 24 (7.1), 47 (P74)
Jeffries K 42 (P55), 45 (P68)
Jenkins A 28 (8.7), 79 (P204)
Jenkinson D 61 (P130), 61
(P132)
Jiang M 60 (P127)
Jodrell DI 35 (P27)
Joel S 78 (P200)
Joel SP 68 (P159), 83 (P220)
Johnson PWM 17 (3.7)
Johnson U 46 (P72)
Johnston D 44 (P64), 46 (P70)
Johnston SJ 19 (4.6)
Jones A 53 (P100)
Jones DJL 81 (P211)
Jones L 48 (P79), 73 (P179)
Jones P 78 (P200)
Jorcano JL 8 (S24)
Judson I 26 (7.6)
Kalas W 57 (P116)
Kan KS 73 (P177)
Kanfer EJ 28 (8.6)
Kanthou C 12 (1.8), 31 (P9)
Kay G 69 (P161)
Keith WN 44 (P63), 55 (P106)
Kelland LR 62 (P136), 65
(P147)
Kellaway J 47 (P73)
Kendrew J 31 (P11)
Kernohan NM 38 (P37), 44
(P64), 60 (P126)
Kerr DJ 1 (CT1), 71 (P169)
Kestelman R 33 (P19)
Khan AA 59 (P124)
Khan N 67 (P153)
Khoo VS 74 (P183), 74
(P184)
Killey J 57 (P113), 57 (P143)
Kirby JA 73 (P178)
Kirkman B 54 (P101)
Knox RJ 70 (P166)
Kobzdej M 57 (P116)
Kofler B 69 (P163)
Koh PK 83 (P218)
Korsmeyer SJ 7 (S18)
Kosuge DD 11 (1.3)
Kote-Jarai Z 24 (7.1), 25 (7.2),
47 (P74)
Kouriefs C 41 (P49), 41 (P51)
Kouzarides T 23 (6.3)
Kumar P 36 (P30)
Kumar S 36 (P30)
Kunkler I 52 (P95)
Kunkler IH 36 (P32)
Kurian KM 25 (7.3), 36 (P32)
Kuschel B 36 (P31)
Kwok M 53 (P97)
Kyung Kang Y 59 (P123)
Lacaine F 1 (CT1)
Laidlaw I 25 (7.5)
Lalani E-N 59 (P124)
Lam E 23 (6.5)
Lambert J 57 (P115)
Lamont A 80 (P205)
Lancashire H 69 (P164)
Landau D 41 (P50), 49 (P84)
Lane D 68 (P158)
Lankester KJ 54 (P104)
Larminie C 23 (6.2)
Laughton C 13 (2.2)
Lawrance J 16 (3.3)
Lawrence L 11 (1.4)
Lawrence NJ 30 (P8), 67
(P153)
Lawry J 55 (P108), 56 (P110),
56 (P111)
Lawton PA 54 (P104)
Layton C 62 (P133)
Le Bricon T 60 (P128)
Leach MO 20 (4.8)
Lebozer C 16 (3.2)
Ledermann JA 19 (4.4)
Lee JR 2 (CT6)
Lee SM 42 (P54)
Leek RD 37 (P34)
Lees NP 81 (P210)
Legge R 51 (P92)
Lemoine N 13 (2.3)
Leonard P 19 (4.4)
Leonard R 37 (P35)
Leonard RCF 39 (P42), 72
(P176)
Leris ACA 41 (P49), 41 (P51)
Leung HY 34 (P22)
Leuratti C 24 (6.8)
Levack P 52 (P95)
Levine T 29 (P4)
Levitt N 78 (P200)
Levy R 3 (S1)
Lewington V 51 (P91)
Lewis C 11 (1.2)
Lewis CE 11 (1.1), 14 (2.6), 59
(P122)
Li L 24 (6.6)
Liang R 7 (S20)
Liddle C 69 (P164)
Lidstone V 50 (P85), 50 (P86)
Liefer KM 8 (S23)
Lillich A 5 (S9)
Lillington DM 16 (3.4)
Lim CK 81 (P211)
Limer JL 55 (P108)
Linch D 1 (CT4)
Link KH 1 (CT1)
Lister A 16 (3.3)
Lister TA 16 (3.4), 84 (P221)
Little MA 64 (P141)
Liu D 14 (2.5)
Liu WM 83 (P220)
Lo SK 22 (5.6)
Loadman PM 68 (P160), 70
(P168)
Lock MR 51 (P90)
Lodge M 12 (1.7)
Loktionov A 45 (P65)
London Gynae-Oncology Group
80 (P206)
Lorigan P 62 (P133)
Lorigan PC 82 (P214)
Lovett B 51 (P90)
Low SE 83 (P219)
Lowdell M 58 (P117), 58 (P118)
Lowe D 28 (8.8)
Lowe SW 4 (S7)
Lu X 15 (2.10)
Lunec J 36 (P29)
Luthra S 30 (P6)
McCall J 45 (P67)
McCarragher LSM 67 (P154)
MacCarthy-Morrogh L 21 (5.5),
56 (P112)
McCartney H 66 (P151)
McClue S 68 (P158)
Author index 87
McConkey C 1 (CT3)
McCready VR 16 (3.2), 72
(P174)
McCulloch TA 11 (1.3)
McDonald JEL 69 (P161)
McGown A 29 (P1)
McGown AT 30 (P7), 30 (P8),
66 (P150), 67 (P153)
McHugh PJ 63 (P139)
McIlhenny C 30 (P5), 40 (P47)
McKay JA 45 (P66)
MacKenzie MAF 25 (7.3), 36
(P32)
Mackie RM 61 (P129)
McKiernan P 72 (P175)
McLean D 57 (P115)
McLees A 23 (6.2)
MacLellan AJ 35 (P27)
MacLennan KA 82 (P216)
McLeod HL 19 (4.6), 35 (P28),
45 (P66), 68 (P157)
MacNeil S 62 (P133)
MacNeill F 25 (7.5)
McNeish IA 28 (8.6), 80 (P208)
McPherson K 53 (P100)
McTiernan A 18 (3.10)
Magee C 60 (P125)
Maheshkumar P 34 (P21), 76
(P189)
Mahmoudi M 79 (P201)
Mainwaring PN 1 (CT4)
Maitland DJ 70 (P168)
Maitland NJ 22 (6.1)
Mak I 31 (P12)
Makris A 37 (P36), 46 (P71)
Mallon EA 40 (P47)
Manson M 20 (4.10)
Margison GP 81 (P210)
Mark MA 35 (P27)
Markham AF 37 (P33), 82
(P216)
Marnett LJ 24 (6.8)
Marra G 17 (3.6)
Marsh H 75 (P187)
Marsh S 35 (P28)
Marshall E 26 (7.7), 42 (P56)
Marshall JF 61 (P130), 61
(P132), 62 (P134)
Marshall S 75 (P187)
Martin A 47 (P73)
Martin J 18 (4.3), 56 (P110)
Martin JE 34 (P21), 76 (P189)
Martin L-A 62 (P135)
Martin SG 21 (5.2)
Martin WMC 43 (P59)
Matheson EC 17 (3.6), 64
(P141)
Mathur P 12 (1.6), 45 (P67)
Matthews J 16 (3.4)
Maughan TS 2 (CT8)
Maxwell RJ 12 (1.7)
Mayalarp SP 66 (P152)
Mayer A 18 (4.2), 32 (P16)
Mead GM 27 (8.5)
Melin C 67 (P156)
Melton RG 70 (P166)
Mendes R 26 (7.8), 43 (P57)
Merritt A 5 (S9)
Meyer T 79 (P201)
Miall S 25 (7.5)
Micallef I 16 (3.3)
Micallef INM 16 (3.4), 84
(P221)
Michael NP 70 (P167)
Michels JE 46 (P69)
Mikaty R 68 (P160)
Miller E 24 (6.6)
Milner J 60 (P127)
Mincher DJ 69 (P161), 69
(P162)
Minto L 64 (P141), 64 (P142)
Minton N 71 (P171), 71 (P172)
Minton NP 70 (P167)
Mistry P 69 (P164)
Mitchell F 19 (4.5)
Mitchell G 25 (7.2)
Moffitt DD 1 (CT1), 76 (P192)
Mokbel K 41 (P49), 41 (P51)
Molife R 62 (P133)
Monaghan JM 34 (P23)
Money-Kyrle JF 53 (P99), 75
(P186)
Moore JV 30 (P7), 77 (P193)
Morgan JG 44 (P61)
Morgan MR 62 (P134)
Morrice J 35 (P25)
Morrison JM 2 (CT6)
Mossman J 53 (P100)
Mostafa A 41 (P49)
Mouzakiti A 21 (5.3)
MPT Collaborators 24 (7.1), 47
(P74)
MRC Bladder Cancer Working
Party 27 (8.2)
MRC Testicular Tumour Group
27 (8.5)
MRC Upper GI Tract Cancer
Group 1 (CT2)
Muir S 55 (P106)
Mukherjee S 49 (P83)
Mulatero C 47 (P73)
Munro AJ 7 (S17)
Murad P 72 (P175)
Murdoch H 70 (P167)
Murray GI 45 (P66)
Murray J 11 (1.3)
Murray JC 13 (2.2), 21 (5.2), 73
(P179), 73 (P180)
Murray LJ 45 (P66)
Nakanishi H 48 (P79)
Nargund VH 34 (P21), 76
(P189)
Naylor MA 31 (P10)
Naylor S 14 (2.6)
Neal DE 73 (P178)
Neale MH 80 (P205)
Neat MJ 16 (3.4)
Nelkin B 24 (6.6)
Nelstrop AE 79 (P201)
Neoptolemos JP 1 (CT1), 13
(2.3), 59 (P123)
Newell DR 65 (P146)
Newlands ES 28 (8.6), 80
(P208)
Newman M 54 (P101)
Nicholl AJ 57 (P113), 57 (P114)
Nicholls E 77 (P195)
Nicholls J 78 (P197)
Nichols J 77 (P196)
Nicoll G 38 (P40)
Nicolson MC 51 (P92)
Nixon SJ 52 (P93)
Noble S 37 (P36)
Nokes L 46 (P69)
Nolan J 31 (P10)
Norman A 74 (P181), 77
(P195)
Norman AR 78 (P197)
Norton A 16 (3.3), 26 (7.8)
Nouri AME 34 (P24), 77 (P194)
Nussey FE 72 (P176)
Nutley BP 68 (P158)
Nutting C 26 (8.1)
Oakley PR 83 (P220)
Oakley RE 14 (2.4)
Oates GD 2 (CT6)
O'Brien M 2 (CT5)
O'Brien ME 42 (P54)
O'Brien MER 26 (7.8), 43
(P57), 43 (P60)
O'Byrne KJ 37 (P34), 42 (P55),
45 (P68)
Ocejo-Garcia M 42 (P53)
O-Charoenrat P 29 (P3)
O'Donnell A 26 (7.6)
Ogilvie DJ 31 (P11)
Okaro A 22 (5.7)
Okaro MC 22 (5.8), 64 (P144)
Okiji S 69 (P163)
Oliver RTD 74 (P182), 77
(P194)
Oliver T 20 (4.9)
O'Malley D 18 (4.3)
Orr S 81 (P211)
Osborne M 46 (P71)
O'Sullivan GC 44 (P61)
Ottensmeier CH 83 (P217)
Ottley CJ 65 (P145)
Overgaard J 8 (S22)
Owen S 16 (3.3)
Packham G 21 (5.3), 21 (5.4),
21 (5.5), 56 (P112)
Padhani A 20 (4.8), 26 (7.6), 26
(7.8)
Pagoulatos C 72 (P173)
Paice E 54 (P102)
Paramio JM 8 (S24)
Parker C 36 (P30), 74 (P181)
Parker CA 59 (P122)
Parker J 72 (P176)
Parry A 65 (P146)
Parry E 44 (P62)
Parry EM 75 (P188)
Parry JM 44 (P62), 75 (P188)
Partridge M 14 (2.4)
Paterson H 25 (7.4)
Payne H 46 (P72)
Payne HA 53 (P99), 75 (P186)
Peacock J 25 (7.4)
Peake D 76 (P192)
Pearson ADJ 36 (P29), 65
(P146)
Pearson DG 65 (P145)
Pederzoli P 1 (CT1)
Pedley RB 11 (1.4), 13 (1.9), 32
(P16), 71 (P170), 71 (P172),
72 (P173)
Perren T 28 (8.7), 50 (P87)
Perren TJ 39 (P44), 54 (P103),
79 (P204)
Perry J 78 (P200)
Perry R 36 (P29)
Pettersson S 69 (P162)
Pettet E 48 (P78)
Pettit GR 30 (P7)
Phillips A 28 (8.7)
Phillips E 14 (2.4)
Phillips H 27 (8.4), 48 (P78)
Phillips JE 54 (P104)
Phillips RM 70 (P168)
Pinto LIB 36 (P29)
Pledge SD 79 (P202)
Plummer R 79 (P203)
Plummer S 20 (4.10)
Plummer SM 24 (6.8)
Pogson CJ 62 (P135)
Pollock JRA 81 (P209)
Ponder BAJ 23 (6.3)
Porteous D 24 (6.6)
Potter BVL 56 (P112)
Povey AC 81 (P210)
Powell MEB 33 (P17), 80
(P206)
Power D 53 (P99), 54 (P102)
Powles J 50 (P88)
Powles TP 25 (7.2)
Pratt NR 44 (P64)
Preece PE 39 (P41)
Price E 65 (P146)
Price P 18 (4.2), 30 (P6)
Prise VE 12 (1.7)
Propper D 78 (P200)
Purdy D 71 (P171)
Purohit A 56 (P112)
Pyle L 31 (P12)
QUASAR Group 1 (CT3)
Quinn H 45 (P67)
Quinn MJ 17 (3.9)
Quirke P 46 (P70)
Rabiasz G 24 (6.6)
Radford J 16 (3.3)
Radstone C 83 (P219)
Radstone CR 78 (P198)
Rampling R 52 (P95)
Raynaud F 26 (7.6)
Raynaud FI 68 (P158)
Rayter Z 25 (7.5)
Reddy A 15 (2.9)
Redfern C 64 (P142)
Reed M 56 (P112)
Rees AR 71 (P171)
Reid M 51 (P92)
Rennison D 67 (P153)
Reznek RH 47 (P73)
Rhys-Evans P 29 (P3), 50 (P88)
Rice K 73 (P180)
Richards F 68 (P159)
Richards M 50 (P85), 50 (P86)
Riches A 39 (P41)
Richman PI 37 (P36)
Robbie IJ 49 (P83)
Roberts JT 27 (8.5)
Robertson H 73 (P178)
Robertson JFR 39 (P43)
Robinson A 2 (CT5)
88 Author index
Robinson C 29 (P2)
Robson J 51 (P89)
Robson L 71 (P170)
Rogers G 22 (5.6)
Rogers PM 65 (P147)
Roggero E 15 (3.1)
Rohatiner A 7 (S19), 16 (3.3)
Rohatiner AZS 16 (3.4), 84
(P221)
Ross G 25 (7.4), 26 (7.7), 41
(P50), 49 (P84)
Ross PJ 13 (2.1)
Ross VG 45 (P66)
Rothman MT 33 (P17)
Routsis DS 75 (P185)
Rowbottom C 26 (8.1)
Rowland IJ 20 (4.8)
Royds JA 55 (P108), 59 (P122)
Rudd R 20 (4.9)
Ruiz S 8 (S24)
Russell P 23 (6.3)
Russell SG 52 (P94), 80 (P207)
Russell ST 81 (P212)
Rustin GJ 12 (1.7)
Rustin GJS 79 (P201)
Rutherford J 15 (2.10)
Ryan A 28 (8.8)
Ryan MP 24 (6.7)
Sacks N 25 (7.5), 41 (P50)
Sagar P 46 (P70)
Sainsbury R 40 (P46)
Samson J 57 (P115)
Samuel K 72 (P176)
Sansom OJ 14 (2.7)
Santos M 8 (S24)
Sargent J 17 (3.8)
Saul MW 57 (P113)
Saunders C 40 (P45)
Saunders M 48 (P77)
Saunders MI 12 (1.5), 27 (8.4),
32 (P15), 43 (P58), 48 (P78)
Sauven P 25 (7.5)
Savory P 20 (5.1)
Sawyer C 61 (P132)
Schapira AHV 22 (5.6)
Schmitt CA 4 (S7)
Scott P 23 (6.2)
Scottish Melanoma Group 61
(P129)
Sebag-Montefiore D 46 (P70)
Seckl MJ 28 (8.6), 80 (P208)
Segrelles C 8 (S24)
Sekhar M 16 (3.5)
Selby P 28 (8.7), 50 (P87)
Sellar G 24 (6.6)
Seymour L 71 (P169)
Seymour M 19 (4.4), 68 (P159)
Shackley D 77 (P193)
Shah EF 40 (P45)
Shah N 12 (1.5), 32 (P15), 43
(P58), 48 (P77), 84 (P223)
Shahbakhti H 18 (4.3)
Shahidi M 77 (P195), 78 (P197)
Shamash J 20 (4.9)
Shankland SH 2 (CT8)
Shanks J 29 (P1)
Sharma RA 24 (6.8)
Sharma SK 13 (1.9), 18 (4.3), 70
(P166), 71 (P170), 71 (P172)
Sharrard RM 22 (6.1)
Sharrocks A 6 (S13)
Shaw J 18 (4.2)
Shedlovsky A 5 (S9)
Shenton K 25 (7.5)
Shepherd J 28 (8.8)
Shepherd P 20 (4.10)
Sherwin KE 33 (P19)
Shoemaker A A 5 (S9)
Shoma AM 59 (P124)
Sibtain A 12 (1.5), 32 (P15), 43
(P58), 84 (P223)
Sierra V 24 (6.7)
Sims EC 33 (P17)
Sims MA 70 (P167)
Singh N 78 (P199)
Singh R 24 (6.8)
Sinnott C 50 (P85), 50 (P86)
Sisley K 36 (P30)
Skelton L 67 (P156)
Skliris GP 37 (P33)
Slater S 20 (4.9), 84 (P221)
Slevin M 20 (4.9)
Slevin ML 47 (P73)
Slevin N 47 (P76)
Slider L 52 (P95)
Sludden JA 79 (P203)
Smalley K 31 (P12)
Smalley KSM 61 (P131)
Smith DB 26 (7.7), 42 (P56)
Smith IE 2 (CT5), 26 (7.8), 43
(P57), 43 (P60)
Smith L 69 (P163)
Smith O 56 (P110)
Smith P 1 (CT4), 15 (3.1)
Smith V 62 (P136)
Smyth JF 24 (6.6), 35 (P27), 61
(P129)
Sodha N 25 (7.2)
Sothi S 47 (P75)
Souberbielle BE 26 (7.8), 43
(P57)
Souhami R 15 (3.1)
Souhami RL 2 (CT7)
Sourvinos G 23 (6.2)
Spanswick VJ 16 (3.5)
Speight PM 62 (P134)
Speirs V 37 (P33)
Spencer DIR 11 (1.4), 71
(P171)
Spencer J 54 (P103)
Spencer L 43 (P57)
Spooner D 1 (CT1), 2 (CT6), 51
(P91)
Springer C 18 (4.3)
Springer CJ 13 (1.9)
Spruce BA 57 (P115)
Staffurth JN 77 (P196)
Stamp GWH 59 (P124)
Statham P 52 (P95)
Stead M 28 (8.7)
Steans G 70 (P168)
Stebbing J 31 (P12)
Steel CM 25 (7.3), 36 (P32), 39
(P41)
Steele IA 34 (P22)
Steele J 20 (4.9)
Stein R 40 (P45)
Stein T 23 (6.2)
Stenning SP 27 (8.5)
Stephens T 65 (P147)
Stephenson TP 75 (P188)
Stevenson FK 83 (P217)
Stevenson GT 73 (P177)
Steward WP 24 (6.8), 45 (P68),
81 (P211)
Stewart AJ 69 (P163)
Stewart LA 2 (CT7)
Stocks SC 44 (P64)
Stoitchkov K 60 (P128)
Stokes ESE 31 (P11)
Strachan SM 24 (6.7)
Strickland S 80 (P208)
Stringer S 29 (P2)
Strzadala L 57 (P116)
Stuart R 44 (P63)
Sue-Ling H 46 (P70)
Sugden E 66 (P149)
Suhr M 48 (P79)
Sumner SG 14 (2.6)
Sutcliffe J 23 (6.2)
Sutton P 26 (7.7)
Sweetenham JW 83 (P217)
Swift I 19 (4.7)
Swift RI 20 (4.8)
Sykes AJ 47 (P76)
Symes M 34 (P24)
Syndikus I 40 (P48)
Tan LT 52 (P94), 52 (P96), 80
(P207)
Tang N 11 (1.2)
Tas MPR 73 (P179), 73 (P180)
Taylor C 17 (3.8), 72 (P175)
Taylor GR 15 (2.10)
Taylor J 32 (P15)
Taylor NJ 12 (1.7)
Tazzyman D 11 (1.2)
Tetlow L 41 (P52)
Thatcher N 42 (P54)
Theti D 67 (P156)
Thliveris A 5 (S9)
Thomas A 42 (P55)
Thomas AL 21 (5.2), 45 (P68)
Thomas GJ 62 (P134)
Thomas HD 79 (P203)
Thomas SJ 33 (P19), 75 (P185)
Thompson A 57 (P115)
Thompson AM 15 (2.8), 38
(P37), 38 (P40), 39 (P41), 44
(P64), 52 (P93), 60 (P126)
Thompson C 34 (P24)
Thompson MJ 23 (6.4), 63
(P137)
Thorp NJ 40 (P48)
Tidy A 25 (7.2)
Tilby MJ 65 (P145), 65 (P146),
66 (P151)
Tilsley DWO 49 (P83)
Tischkowitz M 20 (4.9)
Tisdale MJ 81 (P212), 82 (P213)
Toffa S 58 (P117), 58 (P118)
Toft NJ 14 (2.7)
Tolner B 22 (5.6), 23 (6.5), 70
(P165)
Torabi-Pour N 77 (P194)
Tosh K 23 (6.2)
Townley J 47 (P75)
Townsend PA 21 (5.4), 56
(P112)
Tozer GM 12 (1.7), 12 (1.8), 31
(P9)
Tran G 49 (P81)
TraullE C 15 (3.1)
Trigo J 26 (7.6)
Tsiompanou E 18 (4.3), 29 (P4),
32 (P16)
Turnbull A 69 (P161), 69 (P162)
Turner ML 72 (P176)
Tutt A 25 (7.4)
Tutton MG 19 (4.7)
Twentyman PR 65 (P145)
Uscinska B 18 (3.10)
Uscinska BM 27 (8.2)
Valenti M 32 (P14)
Van Trappen P 28 (8.8)
Vandenberghe EA 82 (P215)
Vandersteen AM 17 (3.7)
Vanhaesebroeck B 61 (P132)
Variend S 56 (P111)
Vaughan Hudson G 1 (CT4)
Vaughan T 70 (P167)
Veal GJ 65 (P146)
Verma N 27 (8.4)
Verschoyle RD 81 (P211)
Vickers E 6 (S13)
Viggers J 54 (P101)
Vini L 50 (P88), 84 (P222)
Vinnicombe S 16 (3.3)
Vlahos I 47 (P73)
Vlatkovic N 60 (P125)
Wakelin D 20 (4.10)
Walker RA 37 (P34)
Wallace DMA 27 (8.3), 76 (P192)
Wallace TW 66 (P152)
Waller ML 30 (P7)
Walsh G 54 (P101)
Wang W 36 (P30), 68 (P157)
Wang X-J 8 (S23)
Ward MA 51 (P89)
Ward TH 66 (P150)
Ward W 73 (P179)
Wardley AM 79 (P204)
Warner TD 33 (P17)
Waterfall M 72 (P176)
Watson M 45 (P65)
Watson R 13 (1.9)
Watt FM 9 (S26)
Watt K 24 (6.6)
Watters A 37 (P35)
Watters AD 76 (P190)
Watters J 65 (P148)
Webb S 26 (8.1), 74 (P183), 74
(P184)
Webley S 18 (4.3)
Webley SD 23 (6.5)
Wedge SR 31 (P11)
Weeden S 18 (3.10)
Weeks B 8 (S23)
Weller I 15 (2.9)
Wells C 41 (P51)
Wells M 11 (1.2)
Welsh K 75 (P187)
West CML 47 (P76)
West Midlands Breast Group 2
(CT6)
Weston M 45 (P67)
Author index 89
Westwood G 58 (P119)
Whawell SA 62 (P134)
Whelan JS 17 (3.9), 18 (3.10)
White JD 11 (1.3), 55 (P105)
White R 23 (6.2)
Whitehurst C 77 (P193)
Whitelegg NR 71 (P171)
Whitney D 75 (P185)
Wigley S 70 (P167)
Wilkie GW 72 (P175)
Wilkins BS 83 (P217)
Wilkinson JM 6 (S14)
Wilkinson PM 27 (8.5)
Williams G 74 (P182)
Williams MV 33 (P19), 51
(P89), 52 (P96)
Williams MW 81 (P211)
Williams N 1 (CT3), 41 (P49)
Williams NJ 41 (P51)
Williams PC 6 (S14)
Williamson C 17 (3.8)
Willingham N 6 (S13)
Wilson C 2 (CT5)
Wilson CB 33 (P19)
Wilson D 13 (2.3), 14 (2.4)
Wilson GA 82 (P215)
Wilson GD 37 (P36), 72 (P173)
Wilson I 12 (1.7)
Wilson P 20 (4.9), 41 (P52), 47
(P73), 74 (P182)
Wims P 27 (8.4)
Winslet M 58 (P117), 58 (P118)
Winter A 23 (6.2)
Winton DJ 14 (2.7)
Withey J 33 (P20)
Witton CJ 38 (P39)
Woll PJ 42 (P53), 55 (P105)
Wood L 21 (5.5)
Woodcock M 72 (P173)
Woodside J 40 (P45)
Workman P 26 (7.6), 32 (P14),
62 (P136), 68 (P158)
Wright JG 79 (P203)
Wright KA 65 (P145)
Yang S-H 6 (S13)
Yates P 6 (S13)
Yiakouvaki A 59 (P121)
Young BD 16 (3.4)
Young IE 25 (7.3), 36 (P32)
Young LS 27 (8.3)
Yu CY 73 (P177)
Zhao S 30 (P7)
Ziyaie D 15 (2.8), 60 (P126)
Zucca E 15 (3.1)
90 Author index

				

